# Type of Speech Material Affects Acceptable Noise Level Test Outcome

**DOI:** 10.3389/fpsyg.2016.00186

**Published:** 2016-02-26

**Authors:** Xaver Koch, Gertjan Dingemanse, André Goedegebure, Esther Janse

**Affiliations:** ^1^Center for Language Studies, Radboud UniversityNijmegen, Netherlands; ^2^International Max-Planck Research School for Language SciencesNijmegen, Netherlands; ^3^Department of ENT, Erasmus Medical CenterRotterdam, Netherlands; ^4^Max-Planck Institute for PsycholinguisticsNijmegen, Netherlands; ^5^Donders Institute for Brain, Cognition and Behaviour, Radboud UniversityNijmegen, Netherlands

**Keywords:** acceptable noise level, speech material type, hearing, working memory, self-control capabilities

## Abstract

The acceptable noise level (ANL) test, in which individuals indicate what level of noise they are willing to put up with while following speech, has been used to guide hearing aid fitting decisions and has been found to relate to prospective hearing aid use. Unlike objective measures of speech perception ability, ANL outcome is not related to individual hearing loss or age, but rather reflects an individual’s inherent acceptance of competing noise while listening to speech. As such, the measure may predict aspects of hearing aid success. Crucially, however, recent studies have questioned its repeatability (test–retest reliability). The first question for this study was whether the inconsistent results regarding the repeatability of the ANL test may be due to differences in speech material types used in previous studies. Second, it is unclear whether meaningfulness and semantic coherence of the speech modify ANL outcome. To investigate these questions, we compared ANLs obtained with three types of materials: the International Speech Test Signal (ISTS), which is non-meaningful and semantically non-coherent by definition, passages consisting of concatenated meaningful standard audiology sentences, and longer fragments taken from conversational speech. We included conversational speech as this type of speech material is most representative of everyday listening. Additionally, we investigated whether ANL outcomes, obtained with these three different speech materials, were associated with self-reported limitations due to hearing problems and listening effort in everyday life, as assessed by a questionnaire. ANL data were collected for 57 relatively good-hearing adult participants with an age range representative for hearing aid users. Results showed that meaningfulness, but not semantic coherence of the speech material affected ANL. Less noise was accepted for the non-meaningful ISTS signal than for the meaningful speech materials. ANL repeatability was comparable across the speech materials. Furthermore, ANL was found to be associated with the outcome of a hearing-related questionnaire. This suggests that ANL may predict activity limitations for listening to speech-in-noise in everyday situations. In conclusion, more natural speech materials can be used in a clinical setting as their repeatability is not reduced compared to more standard materials.

## Introduction

One of the most frequent complaints of adult hearing aid users is that comprehending speech is challenging in noisy environments (Cord et al., 2004; Killion et al., 2004; Nábělek et al., 2006) Indeed insufficient benefit of hearing aids in noisy situations seems to be an important reason for people fitted with a hearing aid not to use it. Hearing rehabilitation could be better attuned to the needs of hearing-impaired individuals if audiologists were able to identify those hearing-impaired individuals who will have problems with accepting higher noise levels in everyday communication situations. Individualized counseling may help hearing-impaired individuals to set realistic expectations of hearing-aid benefit in noise. Furthermore, the use of assistive listening devices could then be applied early on for individuals who can be expected to be unsatisfied with hearing devices in noisy environments in order to ultimately minimize disappointment with the device, activity limitations and participation restrictions related to hearing disabilities (cf. Nábělek et al., 2006; Kim et al., 2015).

This raises the question of how to identify future hearing aid users who may be discouraged from using hearing aids because of difficulty listening in noise. One obvious approach would be to measure the individual’s objective ability to understand speech in noise (e.g., the standard speech-reception threshold measure). However, such objective performance measures are not predictive of hearing aid benefit or success (Bender et al., 1993; Humes et al., 1996; Nábělek et al., 2006). In contrast, one *subjective* measure called “acceptable noise level” or “tolerated SNR” (henceforth, ANL) seems to be predictive of hearing aid and cochlear implant success (Nábělek et al., 1991, 2006; Bender et al., 1993; Humes et al., 1996; Plyler et al., 2008; but cf. Olsen and Brännström, 2014). The ANL procedure involves the following two steps: listeners are first asked to indicate the loudness level they find most comfortable [henceforth, Most Comfortable Loudness Level (MCL), cf. Hochberg, 1975] for listening to a continuous speech signal. In a second step, listeners adjust the background noise level [henceforth, Background Noise Level (BNL)] to the maximum level they are *willing* to put up with while following the running speech presented at their individual MCL level. Subtracting the BNL value from the MCL value yields the ANL measure which typically ranges between -15 and 40 dB with a mean of around 5 to 12 dB (Nábělek et al., 1991, 2006; von Hapsburg and Bahng, 2006; cf. Eddins et al., 2013; Walravens et al., 2014). The lower the ANL value, the more noise the participant accepts while listening to speech. The ANL measure quantifies the individual’s “willingness to listen to speech in background noise” (cf. Nábělek et al., 2006, p. 626). As such, it may be a better indicator of successful hearing aid uptake than the individual’s objective ability to understand speech in noise as it is more telling about the individual’s wishes, motivation, and intentions.

Speech perception is generally considered to involve an interaction between the processing of acoustic information (bottom–up processing) and linguistic and cognitive processing (top–down processing). An important question is how ANL outcome relates to this interaction, as participants are explicitly instructed to ‘follow the speech’ during the ANL task. Even though listeners may engage in setting up linguistic hypotheses about upcoming content when the signal is clear, top–down contextual support may be particularly helpful in reconstructing the message when the signal is presented in noise. It is unclear whether type of speech material affects ANL. The original ANL publications (e.g., Nábělek et al., 1991, 2006) used a standard stretch of read speech, making up a coherent story (the Arizona Travelog passage). In contrast, Olsen and Brännström (2014) used the International Speech Test Signal (ISTS; Holube et al., 2010), which is non-meaningful by definition as the signal consists of roughly syllable-sized units from six different languages and speakers, concatenated into a continuous speech stream. Olsen and Brännström (2014) argue that the ISTS can be used to compare ANL values across languages. However, the use of the ISTS precludes top–down processing. In that sense, the question whether type of speech material affects ANL outcome is a question about the nature of the ANL task in the broader context of models of speech processing. Regarding the question of whether meaningfulness affects ANL outcome, ANLs obtained with unintelligible speech (i.e., reversed or unfamiliar speech) have been found to be higher (i.e., indicative of lower noise tolerance) than those obtained with intelligible speech (Gordon-Hickey and Moore, 2008). In contrast, Brännström et al. (2012a) showed that ANLs were lower for the ISTS in comparison with meaningful speech stimuli. We investigate whether ANL depends on meaningfulness and coherence by using three different stimulus types that differ in meaningfulness (ISTS vs. concatenated sentences and fragments of conversational speech) and coherence (concatenated sentences vs. coherent conversational speech). If meaningfulness of the test material does not affect ANL outcome, listeners’ acceptance of noise while following speech may mainly rely on bottom–up processing. Consequently, following speech in noise as captured by the ANL task would deviate from speech perception and comprehension. In line with Gordon-Hickey and Moore (2008), we expect to find increased ANL values for the non-meaningful ISTS material compared to the meaningful materials. Our hypothesis regarding the direction of a semantic coherence effect is that participants will accept more noise (i.e., show lower ANLs) for the conversational stimulus type in comparison with the passage of concatenated sentences as redundant information is available on the discourse level, which facilitates speech comprehension. Alternatively, however, the faster speech rate and less careful articulation observed in conversational speech may make listening harder than in the sentence materials and may yield lower noise acceptance.

In order for ANL to be a clinically useful tool in hearing rehabilitation, it is important to establish its repeatability (i.e., consistency over repeated measures or test–retest reliability with the exact same materials). Olsen and Brännström (2014) questioned the repeatability of the existing ANL procedures using the ISTS material. In the present study we investigate whether speech material type affects ANL outcomes and repeatability. Relatedly, repetition of the exact same materials may lead to substantial priming effects, especially for the meaningful materials. Consequently, participants would accept more noise upon repeated exposure, yielding a lower repeatability. We investigate whether the use of meaningful materials yields differential repeatability compared to non-semantic ISTS material.

Nábělek et al. (2006) suggest that *future* hearing aid use can be predicted on the basis of ANL outcome for a majority of hearing aid candidates. Olsen and Brännström (2014), however, challenge the predictive value of ANL outcome for hearing-aid use, and report that results regarding the association between ANL and self-reported hearing-aid outcome measures have been mixed. These inconsistent findings may be caused by the multitude of variables that are possibly related to hearing-aid use, hearing-aid satisfaction and hearing-aid success, as reviewed by Knudsen et al. (2010) and McCormack and Fortnum (2013). Note, however, that self-reported hearing problems have been shown to be consistently associated with hearing-aid outcome measures obtained throughout the process of getting a hearing aid (help seeking, hearing-aid uptake, use, and satisfaction). We investigate whether ANL is associated with (specific components of) the Speech, Spatial, and Qualities of Hearing self-report questionnaire (SSQ; Gatehouse and Noble, 2004) and whether this relation depends on ANL test material type. Our expectation is to find differential correlations between the questionnaire outcome and ANL for three speech stimulus types with stronger associations for the more ecologically valid materials.

The central concept of the ANL measure is ‘Listening comfort.’ Thus, individual ANLs are not necessarily linked to the listener’s objective ability to comprehend speech in noise, as shown in a number of studies (cf. Nábělek et al., 2004; Mueller et al., 2006; von Hapsburg and Bahng, 2006; Plyler et al., 2008, but cf. Gordon-Hickey and Morlas, 2015). Whether and how the concept of *comfort* in noisy listening situations relates to *listening effort* is unclear. The clinical meaning of the concept of listening effort has recently been discussed in several papers (McGarrigle et al., 2014; Rennies et al., 2014; Francis and Füllgrabe, 2015; Schulte et al., 2015). One way to quantify *listening effort* is to ask participants to fill in effort-related subscales of self-report questionnaires (cf. McGarrigle et al., 2014). We therefore investigate whether listening effort, as measured with specific questions of the SSQ (Akeroyd et al., 2014) is associated with ANL. We hypothesize that ANL is associated with a listening effort-related subscale of the SSQ with more subjective listening effort related to lower noise acceptance (i.e., higher ANLs).

Listeners need cognitive capacity to map a noisy signal onto stored representations (McGarrigle et al., 2014), as laid out in the Ease of Language Understanding model (Rönnberg et al., 2008, 2013). Multiple studies have shown that hearing aid users’ objective speech understanding in adverse conditions (such as background noise) is related to their working memory capacity, verbal working memory in particular (Akeroyd, 2008; Rudner et al., 2011; Ng et al., 2013, 2014). Given the relatively large amount of unexplained variance for individual ANLs, ANLs may also be associated with working memory. Brännström et al. (2012b) found a significant correlation between working memory capacity and ANL for a sample of normal-hearing participants, with lower noise acceptance (i.e., higher ANLs) relating to poorer working-memory capacity. We investigate whether ANL outcomes obtained with the different types of speech materials relate to listeners’ working memory capacity, where we expect to replicate the results of Brännström et al. (2012b).

As ANL specifically asks listeners about their willingness to accept noise, ANL may be related to personality traits. Indeed, self-control abilities (i.e., the capability to control thoughts, feelings, impulses and performance; Baumeister et al., 1994), have been found to predict ANL outcomes (Nichols and Gordon-Hickey, 2012). We revisit the question to what extent ANL outcome relates to personality characteristics in this study. We expect to replicate effects of self-control on ANL with better self-control related to lower ANLs (cf. Nichols and Gordon-Hickey, 2012). Furthermore, even though earlier studies have not found a link between ANL and age (Nábělek et al., 1991; Moore et al., 2011), nor between ANL and pure-tone hearing thresholds (Nábělek et al., 1991; Freyaldenhoven et al., 2007; Plyler et al., 2007), or between ANL and speech perception accuracy in noise (Nábělek et al., 2004), we investigate whether our data replicate this pattern of results.

This study investigates whether speech material type affects ANL outcomes and repeatability for a reference sample of normal-hearing middle-aged and older participants. As addressing these questions on speech material and repeatability involves relatively long testing sessions with repeated ANL measurements, we tested a non-clinical population first so as not to burden a patient population. Future testing is then required to see whether material type effects generalize to a patient population and whether ANLs based on conversational materials better predict hearing aid success than ANL values obtained with more standard audiology materials (such as, e.g., ISTS).

The present study was set up to address the following four research questions:

(1) Does ANL outcome depend on the meaningfulness (1A) and semantic coherence (1B) of the speech materials?
(2) Does ANL repeatability differ across speech material types?
(3) Are ANLs differentially associated with self-report measures of listening effort and of hearing-related activity limitations for the different speech materials?
(4) Do participant characteristics such as working-memory (4A), and self-control abilities, age, hearing thresholds, and speech perception in noise predict ANL (4B)?

## Materials and Methods

### Participants

Seventy-one adults were recruited, all native speakers of Dutch, above 30 years of age (39 female, 33 male). From the initial sample, we excluded 10 participants whose hearing loss in one or both ears exceeded the Dutch health insurance criterion for partial reimbursement of hearing aids (i.e., pure-tone average over 1000, 2000, and 4000 Hz ≥ 35 dB HL in either ear). We also excluded two participants who suffered from tinnitus and one participant who showed significant binaural low-frequency hearing loss. One participant was excluded because she did not manage to perform the ANL task in the training phase. The 57 remaining participants (34 female, 23 male) ranged in age from 30 to 77 years with an overall mean of 60.7 years (*SD* = 11.0). All participants indicated that they had no hearing impairment and did not use hearing aids. None of the participants had a history of a neurological disease. We followed the protocols of the Radboud University Ethics Assessment Committee for the Humanities. All participants provided written informed consent and were informed that they could withdraw from the study at any time.

### Speech Stimuli

Three types of speech materials were used for ANL testing that differed in meaningfulness and semantic coherence: the unintelligible speech-like ISTS (Holube et al., 2010), a concatenated passage of meaningful Dutch sentences taken from speech material developed by Versfeld et al. (2000; henceforth, SENT), and conversational speech (henceforth, CONV) extracted from the Dutch conversational IFADV corpus (van Son et al., 2008). The 60 s long ISTS signal is made up of units that are roughly syllable sized, originating from six female speakers each reading a short standard passage in their native language (being Mandarin, Spanish, English, German, French, and Arabic). The ISTS signal had been developed on the basis of an automatic procedure to cut, concatenate and reassemble the roughly syllable sized segments from the original six recordings to create a smooth 60 s long speech-like signal including pauses at regular intervals (all pause durations being smaller than 600 ms). The resulting speech rate is approximately 4 syllables per second (Holube et al., 2010). Furthermore, the ISTS signal has been shaped to spectrally match the female international long-term-average speech spectrum (ILTASS, Byrne et al., 1994).

To create the second type of material (SENT), we concatenated fifty sentences from the female speaker of the materials of Versfeld et al. (2000) with intervals of 500 ms silence between sentences (total duration of the passage was 120 s). These sentences are all between five and eight words long and are semantically coherent. A translated example sentence is: “I hope to be able to catch the train.” The speech rate of the sentences ranges between 3.5 to 5.7 syllables per second (Mean = 4.6 syllables/s, *SD* = 0.6). In order to match the spectral properties of the SENT materials to the ISTS materials, the concatenated SENT material was filtered to the ILTASS (combination of male and female signal) using a finite impulse response (FIR) filter between 100 and 16000 Hz.

The third type of speech material was created by extracting two male and two female recordings from the conversational IFADV corpus (van Son et al., 2008). The Dutch open-source IFADV corpus consists of annotated high-quality recording of dialogs on daily topics such as problems in public transport, leisure time activities or vacations. As we wanted to spectrally shape these materials, we selected four longer stretches of speech [CONV1 (female speaker), CONV2 (male speaker), CONV3 (male speaker), CONV4 (female speaker)] where only one speaker was speaking, without being interrupted by the dialog partner. These stretches were based on the available corpus annotations. In a few instances we cut out verbal backchannelling (e.g., “yes,” “hmm”) of the interlocutor, which did not overlap with the target speech. All pauses longer than 500 ms were shortened to 500 ms. The four resulting speech files ranged in duration between 63 and 75 s. Speech rate calculated over the breath groups (sequence of words between inhalations) ranged between 2.6 and 7.5 syllables per second (Mean = 5.7 syllables/s, *SD* = 1.2; CONV1: 6.10 syllables/s, CONV2: 5.10 syllables/s, CONV3: 5.79 syllables/s, CONV4: 5.89 syllables/s). In order to match the spectral contents of the conversational materials to the other types of materials, the four conversational fragments were also filtered to the ILTASS (combination of male and female signal) using a FIR filter between 100 and 16000 Hz.

### Noise Material

The noise stimulus used throughout the ANL test procedure was a non-stationary eight speaker babble noise (BAB8, Scharenborg et al., 2014) filtered to the ILTASS (combination of male and female spectrum) using a FIR filter between 100 and 16000 Hz. In line with the idea of aiming to approximate realistic listening conditions, we used a multi-talker babble noise since it is a typical background sound encountered in daily life.

### Experimental Procedure

#### Test Set-Up

All ANL test materials were presented in a sound-attenuated booth using an Alesis multimix 4USBFX device and Behringer MS16 loudspeakers in front of the listener (0° azimuth) at a distance of 1 m. Stimuli were presented in a custom application (cf. Dingemanse and Goedegebure, 2015) running in Matlab (v7.10.0) on a MacBook Pro (type 9,1). Participants adjusted the sound level of the speech stimuli or the noise file using the up and down keys of a customized keyboard. The starting intensity for the MCL was 45 dB (SPL). The intensity of the speech file for the BNL task was set to the mean of the three measurements in the preceding MCL task. The step size for the intensity adjustment for both tasks was fixed at 2 dB per button press.

All speech and noise materials were scaled to have the same overall level in dB (RMS). Sound level calibration was done using a 2250 Brüel and Kjær real time sound analyzer and a 1000 Hz warble test tone with the same RMS-value as the ANL materials.

#### ANL Instructions

Participants were instructed to first adjust the level of the speech until it was too loud (i.e., up to the first deviation point), then to reduce the intensity until the speech became very soft (being the second deviation point) and lastly find the MCL. Then the participant’s task was to select the maximum BNL they were willing to accept while following the speech at their MCL. They were instructed to use the same pattern of adjustments as described for MCL: turn up the volume of the noise until it was too loud to comfortably listen to the speech (i.e., the first deviation point), then to reduce the noise intensity until the speech became very clear (i.e., the second deviation point) and lastly to find the maximal background noise level they were willing to put up with while following the speech signal (BNL).

#### Familiarization Phase

In order to familiarize participants with the ANL procedure prior to actual testing, each participant was presented with a phonetically balanced Dutch training fragment. A 2-min-long recording of a female Dutch speaker reading a standard text passage (*Dappere fietsers* – ‘Brave cyclists’) served as training material. The noise stimulus (BAB8) used throughout the actual ANL test (BNL part) also served as background noise during the training session. Participants first received written instructions on the experimental task (which was a Dutch translation of the instruction provided in Nábělek et al., 2006, p. 639). The experimenter then demonstrated the task, using scripted instructions, which again followed the translation of Nábělek et al. (2006). A visual display was available during the familiarization phase that enabled the participant, as well as the experimenter, to see the course of the presentation level during the MCL and the BNL tasks. Each participant had to demonstrate the expected intensity pattern (up-down-final adjustments, cf. deviation points above) three times in a row for both MCL and BNL components before they could proceed with the test phase.

#### Test Phase

Unlike during the familiarization phase, visual output was available only to the experimenter during the ANL test sessions. Participants had to perform the MCL and BNL tasks for each of the six ANL test stimuli, and each of the two tasks was repeated three times in a row to decrease measurement error (cf. Brännström et al., 2014b; Walravens et al., 2014). The ANL for each fragment and for each participant was calculated by subtracting the mean BNL from the averaged MCL. Note that stimulus presentation was looped such that if participants had not provided their response before the end of the stimulus, the stimulus was automatically repeated. All participants managed to set the MCL and BNL levels within the stimulus duration in the test phase (minimal duration: 60 s for the ISTS).

#### Test Repetition

In order to test the repeatability of the ANL measures across the different materials, we asked the participants to do the ANL task twice for each stimulus type (ISTS, SENT, CONV) with exactly the same material. Note that we took into account that the repetition of the exact same materials across sessions could lead to substantial priming effects, especially for the meaningful materials, by including a control variable in our models to capture changes in ANL over test sessions. Participants first performed the ANL test with the different materials at the beginning of the test session, and again (approximately 1 h later) toward the end of the session. Participant characteristics data were collected in between these two ANL test sessions. During the first ANL session (session I), six different fragments were presented: ISTS, SENT, CONV1, CONV2, CONV3, and CONV4. To restrict testing time, we only presented one fragment for each of the three material types in the test repetition (session II): ISTS, SENT, and CONV4. We selected the CONV4 stimulus from the four conversational test fragments because it featured a female speaker (as was the case for the ISTS and the SENT material) and because its speech rate was typical for conversational speech (i.e., 5.89 syllables per second).

#### Randomization

We used a block-wise randomization procedure to minimize presentation order effects for the material types. Each participant was pseudorandomly assigned to one out of six possible block orders for the speech material types (ISTS, SENT, CONV). The order of the presented speech material types for the second test session (session II) matched the order of session I.

The order in which the four conversational materials appeared in the first ANL test session was also randomized. Each participant was randomly assigned one out of 24 possible presentation orders for the conversational speech stimuli.

### Tests of Participant Characteristics

#### Hearing (Pure-Tone Average)

**FIGURE 1:**
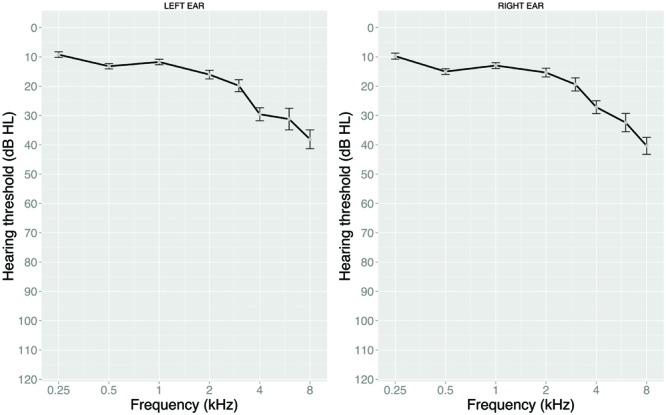
**Mean audiometric pure-tone air conduction thresholds (for left and right ear) as a function of frequency.** Error bars represent standard errors.

Hearing status was screened with air conduction pure-tone audiometry using the modified Hughson-Westlake technique for octave-frequencies between 250 and 8000 Hz, including two half-octave frequencies of 3000 and 6000 Hz (see **Figure 1**). Audiometric averaged thresholds were calculated for the better ear as auditory presentation of the ANL test was binaural. Seven participants showed an asymmetric hearing loss, defined as an interaural difference of more than 10 dB averaged over 500, 1000, 2000, and 4000 Hz (Noble and Gatehouse, 2004). In addition to the pure-tone average over 1000, 2000, and 4000 Hz, we calculated high-frequency PTA^HF^ as the mean threshold over 3000, 4000, 6000, and 8000 Hz. **Table 1** displays descriptives for the two PTA measures. Higher values indicate poorer hearing.

**Table 1:** Descriptives for the participant characteristics.

	*M*	*SD*	Range
Age (years)	60.72	11.04	30–77
PTA (dB HL)	16.05	8.16	0–31.67
PTA^HF^ (dB HL)	25.09	15.68	-1.25–56.25
Speech perception in noise (% correct)	88.22	6.79	67.88–96.36
Reading Span (% correct)	28.43	10.73	0–48.15
Self-Control Scale (% of maximum)	67.34	12.05	38.46–93.85
SSQ Part 1 ‘Speech hearing’ (mean score)	7.07	1.07	4.86–9.36
SSQ Part 3 ‘Qualities of hearing’ (mean score)	7.98	0.93	5.50–9.83
SSQ ‘effort and concentration’ (mean score)	6.55	1.71	3.00–9.50

#### Speech Perception in Noise

Speech perception in noise was tested using a standard Dutch speech audiometry test, the CVC word material from Bosman and Smoorenburg (1992, 1995), which is common in clinical practice in the Netherlands. The test allows presenting the materials at SNRs which are reasonably representative of noise levels during everyday communication (Smeds et al., 2015). This test material consists of meaningful monosyllables (e.g., *kaas*, ‘cheese’) produced by a female speaker arranged in lists of 12 words. The material was presented in a sound-attenuated booth using Behringer MS16 loudspeakers placed in front of the listener (0° azimuth) at a distance of one meter. The CVC words were presented at an intensity level of 65 dB (SPL) mixed with a masking noise of the same intensity (long-term-average spectrum of the recorded speaker). The test score was based on the number of correctly reproduced phonemes (max. three per test item), discarding the first item of each list (which is considered a practice item). Based on Bosman and Smoorenburg’s standardizations results, we expected a mean phoneme accuracy score of about 80–85% for normal hearing adult participants at an SNR of 0 dB (more favorable signal-to-noise ratios may thus lead to ceiling effects in performance). All participants were presented with five consecutive lists (list 31–35), which resulted in a maximum accuracy score of 165 phonemes correct (5 lists × 11 items × 3 phonemes). The speech perception in noise score reported here was quantified as the percentage of correct phonemes produced. **Table 1** provides the descriptives for the perception in noise score. Higher values indicate better speech perception in noise.

#### Reading Span

We used a Dutch version of the well-established reading span test to index working memory (cf. Daneman and Merikle, 1996; Besser et al., 2013; Besser, 2015, Unpublished). The Dutch test consists of 54 grammatically correct sentences, consisting of a noun phrase plus verb phrase. The 54 sentences are divided in 12 sets of three, four, five, or six consecutive sentences. Half of the 54 sentences make sense (e.g., The student sang a song); the other half is absurd (e.g., The daughter climbed the past). The sentences were presented orthographically in chunks: first the subject noun phrase was presented (determiner-noun, e.g., *The student*), followed by the verb (e.g., *sang*), followed by the object noun phrase (determiner-noun, e.g., *a song*; cf. Besser, 2015, p. 173). We used E-prime (2.0, Psychology Software Tools) to present the chunks of the respective test sentences (Subject, Verb, and Object) consecutively on a computer screen (display time of each chunk: 800 ms, blank inter chunk interval: 75 ms). Font size was 36 pt (Verdana). The primary unspeeded task was to repeat back either the first or the last nouns of the respective test set ranging in length from three to six consecutive sentences. Thus, participants were visually prompted to (orally) recall either the subject noun phrases (first nouns) or the object noun phrases (last nouns) of the 12 test sets. The order in which participants recalled the first or last words was not taken into consideration for the scoring (cf. Besser et al., 2013). Additionally, participants were asked to perform a speeded plausibility judgment after each sentence as a secondary task. This task ensured that participants read and comprehended the sentences. Response time was restricted by imposing a time out of 1.75 s after a visual prompt appeared that initiated the plausibility judgment task. Participants gave their plausibility judgment by either pressing a red (i.e., absurd) or a green button (i.e., makes sense) on a customized standard keyboard. Participants received written task instructions and completed a training test set before the actual test started. Reading span score was quantified as the percentage of correctly recalled nouns across the 12 sets. **Table 1** displays the descriptives for the Reading Span test. Higher values indicate better working memory capabilities.

#### Self Control

Participants filled in a Dutch translation of the Brief Self-Control Scale, a 13 items questionnaire using a five-point Likert scale (Tangney et al., 2004; cf. Kuijer et al., 2008). Individual test score were quantified as the percentage of points out of the maximum of 65 points. **Table 1** displays the descriptives for the self-control predictor variable. Higher values indicate better self-control abilities.

#### SSQ Questionnaire

Prior to the ANL testing session, participants filled in an online (Dutch) version of the Speech, Spatial and Quality of Hearing Scale (SSQ, Gatehouse and Noble, 2004). The SSQ self-report scale, which consists of 49 items, is subdivided into three parts: Part 1: ‘Speech hearing’ (14 questions), Part 2: ‘Spatial hearing’ (17 questions), and Part 3: ‘Qualities of hearing’ (18 questions). Following Akeroyd et al. (2014), we extracted a factor related to listening effort covering question numbers 15 and 18 of the SSQ subscale ‘Qualities of hearing’ (‘Do you have to put in a lot of effort to hear what is being said in conversation with others?’; ‘Can you easily ignore other sounds when trying to listen to something?’). Hence, we calculated the SSQ ‘effort and concentration’ subscale by averaging scores over these two questions. We also calculated the average over the first and the third SSQ scale as these two were deemed most relevant. **Table 1** presents the descriptive values for averaged SSQ ‘Speech hearing’ and ‘Qualities of hearing’ scores, as well as for the factor related to listening effort (SSQ ‘effort and concentration’). Higher values on the SSQ scale indicate fewer limitations in self-reported activity due to hearing problems. **Table 2** provides a correlation matrix of all the participant-related characteristics.

**Table 2:** Correlation matrix with correlation coefficients and significance levels for participant characteristics (Spearman’s rank, uncorrected).

	Age	PTA^HF^	Speech perception in noise SPIN	Reading span RST	Self-control scale SCS	SSQ ‘Speech hearing’ SSQ^1^	SSQ ‘Qualities of hearing’ SSQ^3^	SSQ ‘effort and concentration’ SSQ^EC^
Age								
PTA^HF^	0.42^∗∗^							
SPIN	-0.48^∗∗∗^	-0.71^∗∗∗^						
RST	-0.35^∗∗^	-0.28^∗^	0.51^∗∗∗^					
SCS	0.08	0.07	0.01	-0.06				
SSQ^1^	-0.19	-0.08	0.22.	-0.03	0.39^∗∗^			
SSQ^3^	-0.17	0.01	0.21	-0.06	0.39^∗∗^	0.65^∗∗∗^		
SSQ^EC^	-0.10	-0.07	0.17	-0.02	0.34^∗∗^	0.54^∗∗∗^	0.64^∗∗∗^	

*Significance level notation: ^∗∗∗^*p* < 0.001; ^∗∗^*p* < 0.01; ^∗^*p* < 0.05; .*p* < 0.1.*

### Analyses

#### RQ1

Two separate statistical regression models were run to investigate the effects of meaningfulness and coherence (RQ1) of the test material on ANL, using linear mixed-effect models with participants as random variable. The program R was used with the lme4 package (Bates et al., 2013) and restricted maximum likelihood estimation. *p*-values were calculated using the ANOVA function of the car package which calculates type II Wald χ^2^ values. The categorical within-subject variable *meaningfulness* included two levels: not meaningful (ISTS material) vs. meaningful (CONV and SENT material). The within-subject variable *coherence* featured two categories: coherent on sentence level (SENT material) vs. coherent on discourse level (CONV material). Block order (order a–f) was included as additional control variable in all models. For the model on meaningfulness (model 1A), we allowed for the possibility that the effect of meaningfulness differed across participants by including a random participant slope for meaningfulness. Similarly, we allowed for the possibility that the effect of semantic coherence differed across participants by including a random participant slope for meaningfulness in the ‘coherence’ analysis (model 1B). Note that we also included the interaction between session number and meaningfulness (in model 1A) or between session number and coherence (in model 1B), to allow for the possibility that ANLs may systematically change with session number due to semantic priming. Consequently, we also allowed for the possibility that the effect of session number differed across participants by including a random participant slope for both models (model 1A, model 1B).

#### RQ2

We first ran a linear mixed-effect model (with random intercepts for participants) with ANL differences between test sessions as dependent variable. The question was whether ANL values obtained for the three types of speech materials differed in their repeatability across test sessions. One outlier was excluded from repeatability analysis of the ISTS material as the ANL difference between sessions I and II of this participant exceeded a threshold of the sample mean plus three standard deviations.

Apart from the mixed-effect analysis described above, we followed the procedures described by Brännström et al. (2014b) to assess the repeatability of the three speech materials. Hence, we inspected the Bland–Altman plots (Bland and Altman, 1986; Vaz et al., 2013) as well as the coefficient of repeatability (henceforth, CR) for each of the three test materials for which two test sessions had been run. The CR measure is a repeatability (test–retest reliability) measure. It indicates the size of the measurement error in its original measured unit (i.e., dB). In our case, it represents the size of the difference between one measurement (session) and another measurement using the exact same material (with 95% confidence level). The Bland–Altman plots show for each of the three speech materials (ISTS, SENT, CONV4) each participant’s mean ANL over the two sessions on the x-axis against the difference between the two sessions on the y-axis. The CR was calculated for each material by multiplying the standard deviation of the differences between ANLs (averaged over repetitions) for the two sessions with 1.96. Additionally, we calculated the coefficients of repeatability for all test materials (i.e., incl. CONV1, CONV2, and CONV3) over their three repetitions within test sessions (repetition 1 vs. repetition 2; repetition 2 vs. repetition 3). This enabled us to analyze whether repeatability changed within and across test sessions.

#### RQ3

To assess the question whether self-reported hearing related activity limitations and listening effort differentially predict ANL outcomes for the three different speech materials (RQ3) we set up four linear mixed-effect models that included a categorical speech material variable (ISTS, SENT, CONV) in interaction with one of three variables derived from the SSQ scale (SSQ Part 1, SSQ Part 3, SSQ ‘effort and concentration’). Session number was added as categorical covariate to capture repetition effects due to semantic priming. Again, we allowed for the possibility that the effects of session number and speech material differed across participants and therefore added random slopes for the variable speech material and session number to the model.

#### RQ4

To investigate the effects of participant characteristics (age, hearing thresholds, speech perception in noise accuracy, working memory, and self-control abilities) on ANL for the three speech materials (RQ4) we performed 15 correlation analyses (Pearson’s *r*) and Bonferroni corrected for multiple comparisons. ANL values were pooled across the two test sessions.

## Results

**FIGURE 2:**
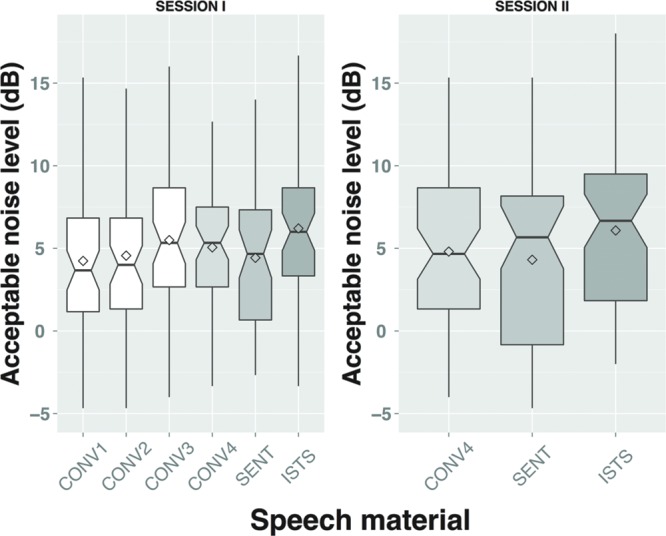
**Acceptable noise level (ANL) test results per speech material and per test session.** Note that the notch plots include a marker for the mean (diamond symbol).

**Table 3** shows the ANL test results per speech material per test session for the three unrepeated conversational materials (CONV1-3) and the three repeated materials (CONV4, SENT, ISTS). Mean ANLs are higher for the ISTS material than for the meaningful materials. **Figure 2** gives an overview of the ANL test results per test session including the conversational materials that were only presented in test session I (i.e., CONV1, CONV2, and CONV3).

**Table 3:** Acceptable noise level (ANL) descriptive statistics for the six speech materials and the two test sessions (in dB).

Test material	Test session I		Test session II	
	*M*	*SD*	*M*	*SD*
CONV1	4.06	4.59	–	–
CONV2	4.39	4.58	–	–
CONV3	5.50	4.29	–	–
CONV4	5.30	4.43	4.81	4.53
SENT	4.32	5.57	4.13	5.24
ISTS	6.25	4.90	5.84	5.25

### Research Question 1A: Does ANL Outcome Depend on the Meaningfulness of the Speech Material?

The results of the statistical model (cf. **Table 4**) showed that ANLs for the meaningful materials (SENT, CONV) were significantly different from those for the non-meaningful ISTS material [χ^2^(1, *N* = 341) = 17.98, *p* < 0.001]. Participants showed 1.46 dB higher ANLs and thus less noise acceptance for the ISTS signal in comparison with the meaningful materials. The observed effect direction matched our *a priori* hypothesis that participants would accept less noise for the non-semantic ISTS material than for the meaningful materials. Block order of presentation did not influence ANL, nor did session number. These control variables also did not interact with the meaningfulness of the test material. The absence of a significant effect of session number on ANL suggests that ANL was stable over sessions and that no semantic priming occurred between sessions. This absence of priming held across material types as the meaningfulness × session number interaction was insignificant. Block order did not affect the ANL outcome, which suggests that our randomization procedure was adequate. For reasons of brevity block order is left out in the model presented below [the variable having six levels; χ^2^(5, *N* = 341) = 2.13, *p*> 0.1].

**Table 4:** Model testing for the effect of meaningfulness on ANL.

	Estimate	*SE*
Intercept	4.79	0.62
Meaningfulness	1.46	0.44^∗∗∗^
Session number	-0.32	0.34^ns^
Meaningfulness × session number	-0.09	0.59^ns^

*Significance level notation: ^∗∗∗^*p* < 0.001; ^ns^*p* > 0.1.*

We also investigated the effect of meaningfulness including all conversational materials (this implies that it can only be assessed for session I). To that end, we averaged ANLs per participant over the conversational materials (CONV1–CONV4). In line with the results presented in **Table 4**, this analysis showed an effect of meaningfulness on ANL with less noise acceptance for the non-meaningful ISTS material compared to the two types of meaningful materials [χ^2^(1, *N* = 170) = 18.47, *p* < 0.001].

### Research Question 1B: Does ANL Outcome Depend on the Semantic Coherence of the Speech Material?

A significant effect of coherence was observed with higher ANLs for the material with coherence on discourse level, i.e., the conversational material [χ^2^(1, *N* = 227) = 6.04, *p* < 0.05] than for the concatenated sentences (cf. **Table 5**). Thus, for the conversational test material participants accepted less background noise. The size of the effect was 1.05 dB. The observed direction of the effect matched the hypothesis that participants would accept less noise for the conversational material, which was coherent at the discourse level, but may have been more difficult in terms of speech rate and speaking style than the concatenated sentences. Again, neither simple nor interaction effects (with the variable of interest, i.e., coherence) were found for the predictors session number and block order suggesting that the randomization procedures were appropriate and that there was no semantic priming from the first to the second session. The control variable block order is not included in the model below for reasons of brevity [χ^2^(5, *N* = 227) = 2.62, *p*> 0.1].

**Table 5:** Model testing for the effect of semantic coherence on ANL.

	Estimate	*SE*
Intercept	4.25	0.72
Coherence	1.05	0.46^∗^
Session number	-0.12	0.43^ns^
Coherence × session number	-0.37	0.60^ns^

*Significance level notation: ^∗^*p* < 0.05; ^ns^*p* > 0.1.*

We also investigated whether the coherence effect can be generalized to different conversational speech fragments by replacing the conversational ANL values in the analysis above (CONV4) by the average ANL over the four conversational speech materials (CONV1–CONV4) per participant (for the first session only). The results of this alternative analysis did not replicate the previous finding of a coherence effect on ANL [χ^2^(1, *N* = 113) = 1.41, *p*> 0.1]. Thus, there is no clear evidence for a coherence effect on ANL in our data. We raised the possibility that speech rate may affect ANL outcomes and that the difference between the conversational and concatenated sentences material is not just about discourse coherence, but also about speech rate. To follow up on that, we tested whether speech rate differences between the four conversational fragments affected ANL outcome by setting up a linear mixed-effect model with speech rate as a continuous predictor of ANL (first session measurements only, only conversational fragments). Speech rate turned out not to be a significant predictor of ANL in this subset analysis [χ^2^(1, *N* = 228) = 0.33, *p*> 0.1].

### Research Question 2: Does ANL Repeatability Differ Across Speech Material Types?

**FIGURE 3:**
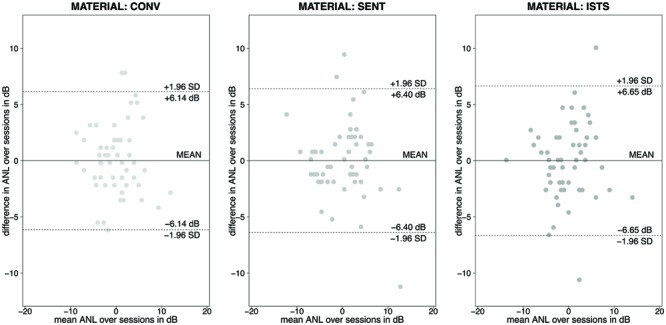
**Bland–Altman plots for repeated ANL tests using conversational (CONV), concatenated sentence (SENT) and ISTS material.** Horizontal lines represent the mean of the differences over the two test sessions as well as the boundaries for the 95% confidence interval per material type.

The mixed-model analysis did not show a significant speech material effect on repeatability of the ANL, quantified as the difference between the ANLs per participant for the two test sessions [χ^2^(2, *N* = 169) = 0.57, *p*> 0.1]. In an additional analysis on repeatability across material types we used the statistical approach of the *coefficient of repeatability* (CR). **Figure 3** displays the Bland–Altman plots for the three materials for which two test sessions had been run.

The highest coefficient of repeatability and thus the lowest repeatability was found for the ISTS material (CR = ± 6.65 dB). Both the concatenated sentences material (SENT) as well as the conversational material showed lower coefficients of repeatability and thus numerically slightly better repeatability. For the concatenated sentences material (SENT) the CR was ±6.40 dB. The best repeatability (numerically) was found for the conversational test material with a CR of ±6.14 dB. The combination of these two analyses suggests comparable repeatability across the speech materials.

In an additional step we calculated the coefficients of repeatability for all test materials over subsequent repetitions within test sessions. **Table 6** shows that ANL repeatability increased numerically (i.e., CRs decreased) within test session I for all test materials except for CONV3. The same pattern of improved repeatability is seen for the CRs within test session II except for the SENT material. Overall, the repeatability in test session II does not seem to be numerically different from the repeatability in test session I. Note that repeatability seems to be most stable for the CONV4 material both within and across test sessions.

**Table 6:** Coefficients of repeatability (in dB) for ANL for the six speech materials and the two test sessions contrasting subsequent repetitions.

Test material	Test session I		Test session II	
	Repetition 1 vs. 2	Repetition 2 vs. 3	Repetition 1 vs. 2	Repetition 2 vs. 3
CONV1	6.04	4.42	–	–
CONV2	6.87	5.29	–	–
CONV3	5.76	6.34	–	–
CONV4	4.98	4.75	5.50	5.07
SENT	6.38	4.65	4.32	6.06
ISTS	6.76	4.68	6.16	5.76

### Research Question 3: Are ANLs Differentially Associated with Self-Report Measures of Listening Effort and of Hearing-Related Activity Limitations for the Different Speech Materials?

We first tested whether the first *subscale* of the SSQ self-report questionnaire (‘Speech hearing’) would be associated with ANL outcomes. The model showed significant material effects [χ^2^(2, *N* = 341) = 21.39, *p* < 0.001] with highest ANLs found for the ISTS material and lowest ANLs for the sentence material (SENT). Importantly, this model showed a significant effect of the subjective questionnaire predictor SSQ (subscale ‘Speech hearing’) on ANL [c^?2^(1, *N* = 341) = 4.62, *p* < 0.05, see **Table 7**]. Higher scores on the SSQ subscale (i.e., fewer self-reported limitations due to hearing problems) were associated with more noise acceptance and thus lower ANLs. For an increase of 1 point on the SSQ ‘Speech hearing’ subscale the model predicted an ANL decrease of approximately 1 dB, which corresponds to an overall effect size of 4.4 dB (with the SSQ ‘Speech hearing’ subscale ranging from 4.86 to 9.36). However, the model did not show differential SSQ subscale effects on ANL for the three materials [χ^2^(2, *N* = 341) = 0.74, *p*> 0.1].

**Table 7:** Model testing for differential associations between SSQ subscale scores and ANLs for three speech materials (CONV, SENT, ISTS).

	Estimate	*SE*
Intercept (CONV material)	12.14	3.65
SENT material	-2.73	2.36ˆns
ISTS material	0.97	2.39ˆns
SSQ Part 1 (‘Speech hearing’)	-0.98	0.51ˆ*
Session number	-0.34	0.31ˆns
SSQ (‘Speech hearing’) × SENT material	0.26	0.33ˆns
SSQ (‘Speech hearing’) × ISTS material	0.003	0.33ˆns

*Significance level notation: ^∗^*p* < 0.05; ^ns^*p* > 0.1.*

We also investigated the association between the third *subscale* of the SSQ self-report questionnaire (‘Qualities of hearing’) and ANL. The model showed significant material effects with lowest ANLs for the sentence material [χ^2^(2, *N* = 341) = 21.31, *p* < 0.001]. However, we did not find an association between ANL and the third *subscale* of the SSQ self-report [χ^2^(1, *N* = 341) = 0.43, *p*> 0.1], nor differential SSQ ‘Qualities of hearing’ effects on ANL for the three materials [χ^2^(2, *N* = 341) = 1.56, *p*> 0.1].

In a third step we analyzed the association between the factor ‘Effort and concentration’ (questions number 15 and 18 of the ‘Qualities of hearing’ *subscale* of the SSQ) and ANL. As for the analyses above, the model showed significant material effects with lowest ANLs for the sentence material [χ^2^(2, *N* = 341) = 21.32, *p* < 0.001]. Yet, neither an association of ANL with the factor ‘Effort and concentration’ [χ^2^(1, *N* = 341) = 1.80, *p*> 0.1] nor differential ‘Effort and concentration’ effects on ANL for the three materials were found [χ^2^(2, *N* = 341) = 1.30, *p*> 0.1].

Additionally, we explored the strength of the association between the SSQ self-report measures (subscale ‘Speech hearing’) and the ANLs (pooled over sessions) separately for the three materials by running correlation analyses. Only for the conversational material (CONV) a marginally significant correlation (*r*= -0.23, *p*= 0.082, Pearson’s *r*) was found.

### Research Question 4: Do Participant Characteristics such as Working Memory (4A), and Age, Hearing Thresholds, Speech Perception in Noise, and Self-control Abilities Predict ANL (4B)?

Again, ANLs were pooled over the two test sessions for each of the three materials. Working memory was not correlated with ANL (*p* > 0.1). Likewise, none of the other correlations (*N* = 15) were statistically significant at an alpha level of 0.05 (i.e., not even before application of any correction required for multiple testing). Similarly, adding participant characteristics as continuous variables to either of the linear mixed-effect models discussed above (for research questions 1A and 1B) did not yield any significant effects of these participant-related variables.

## Discussion

The clinical purpose of the ANL test is to predict self-reported hearing problems and future hearing aid success as reliably as possible. Therefore, it is crucial to know whether and how its clinical applicability depends on what speech material listeners are presented with and how the test is administered. Material effects on the outcome of the ANL test have been addressed in numerous studies (von Hapsburg and Bahng, 2006; Gordon-Hickey and Moore, 2008; Olsen et al., 2012a,b; Ho et al., 2013; Olsen and Brännström, 2014). In a number of recent publications (Brännström et al., 2012a, 2014a,b; Olsen et al., 2012a,b) – the ISTS (Holube et al., 2010) has been used, which is non-meaningful by definition. However, the original ANL test fragment used by Nábělek et al. (2006), in which ANL outcome was shown to be predictive of hearing aid uptake, was a meaningful and coherent read story, and thus linguistically different from the ISTS material. With the present study we investigated material effects on ANL to find out whether meaningfulness and coherence affect ANL (RQ1). In addition, we evaluated the repeatability of the ANL test across a range of test materials to check whether ecologically more valid materials yield a comparable repeatability as more standard audiology materials and the ISTS signal (RQ2). Further, we analyzed the association between ANLs and the outcome of a questionnaire that measures activity limitations due to hearing problems to elaborate on the connection between listening effort and ANLs. We also re-examined the association of working memory and self-control abilities and ANLs (RQ4) found in previous studies (Brännström et al., 2012b; Nichols and Gordon-Hickey, 2012).

As expected, ANLs were higher for the ISTS material in comparison with the meaningful materials. Our interpretation of this effect is that the available redundancy for the meaningful materials facilitated speech processing (via top–down processing) and thus led participants to choose higher levels of acceptable noise (i.e., lower ANLs) than for the non-meaningful material. The unintelligible ISTS signal might have led participants to still want to hear as much as possible (i.e., relying more heavily on bottom–up processing). Furthermore, contrasting conversational ANL test materials with a passage of concatenated standard audiology sentences, we have not found convincing evidence for a semantic coherence effect on ANL. Possibly, the faster and more casual speaking style in the conversational material made listening more difficult, but this speaking style effect may have been offset by greater semantic coherence in the conversation, providing a form of discourse redundancy. The data did not provide clear evidence for priming effects across tests sessions (but note that **Table 6** shows that coefficients of repeatability were largest between the first and second measurement within test session I). All in all, these results provide some evidence that top–down processing plays a role in ANL performance.

An important question was whether repeatability differs across the three speech materials. Neither the statistical modeling approach nor the analysis of the coefficient of repeatability (CR) showed statistically differential repeatability. Rather, repeatability was comparable for the three speech material types with CR values ranging between ±6.14 dB for the conversational material and ±6.65 dB for the ISTS material. Crucially, a coefficient of repeatability lower or equal to ±6 dB ensures that measurement error is lower than the distance between the two thresholds used to categorize hearing aid users as either successful or unsuccessful (≤7 and >13 dB, cf. Nábělek et al., 2006). Across test sessions, all three speech material types yielded CRs just above the critical ±6 dB threshold. With respect to ANL repeatability within test sessions, the conversational material (CONV4) yielded most stable CRs with values below ±6 dB. Our interpretation of the relatively high CR values across sessions is that listeners’ internal criteria for MCL and BNL may be somewhat variable over time, particularly if they are engaged in other activities in-between test and retest measurements. As suggested by Brännström et al. (2014b), noise acceptance while following speech may best be considered a range (Acceptable Noise Range), rather than a specific level (ANL). The relatively poor repeatability of ANL may raise concerns about the clinical value of the ANL as an indicator for hearing aid use and success. However, if the ANL is used to compare two hearing aid conditions within one session, within-session reliability seems to be sufficient. For example, the ANL has been used successfully to show the effect of a noise reduction algorithm (Mueller et al., 2006; Peeters et al., 2009; Dingemanse and Goedegebure, 2015). Further research would be required to investigate whether Acceptable Noise Range may be a more reliable predictor of hearing problems and future hearing aid success than ANL.

Our analysis on the association of ANLs and the outcome of a subjective hearing-related questionnaire (RQ3) relates to recent discussion about the clinical meaning of concepts such as listening effort and fatigue in hearing-impaired individuals (McGarrigle et al., 2014). Our data showed a significant effect of participants’ score on the subscale ‘Speech hearing’ of the Speech, Spatial, and Qualities of Hearing self-report (SSQ, Gatehouse and Noble, 2004) on ANL, particularly when listening to conversational speech. Participants who reported fewer listening problems also tolerated more noise while listening to speech (i.e., lower ANLs). Most questions of the ‘Speech hearing’ subscale are about conversation in noise. Both measurements (SSQ and ANL) are subjective judgments, where SRT measurements are not. This makes an association between ANL and SSQ more likely than an association between SRT and SSQ. The subscale ‘Qualities of Hearing’ was not significantly correlated with ANL. The between-participant differences of the ‘quality of sound rating’ were relatively small in this group of nearly normal-hearing participants. Possibly, perceived sound quality and ANL may be associated among hearing-impaired participants. No association was found between ANL and the subscale ‘Effort and Concentration.’ This suggests that noise tolerance (as one aspect of listening comfort), is a different concept than the listening effort concept as formulated in these specific questionnaire questions. Further research should clarify differences and commonalities of both concepts.

The association between self-reported listening difficulties in noise and noise acceptance (i.e., ANL) only becomes evident when such an ANL test relates to everyday experiences. We think this result clearly makes a case for the use of ecologically valid conversational materials in clinical testing. Audiologists and speech researchers should think about how representative the type of noise and noise levels are of everyday listening, but they should also care about differences between read aloud speech and spontaneous conversation.

Further, the attempt to replicate working memory effects on ANL was unsuccessful. This suggests that noise tolerance, as one aspect of listening comfort, is not related to individual working memory capacity. Importantly, in line with previous studies (cf. Akeroyd, 2008), working memory was considerably correlated with speech perception in noise (cf. **Table 2**), with higher working memory relating to better speech perception. The failure to replicate working memory effects on ANL in our study can be accounted for in two ways. First, it may be due to the use of different test materials and test procedures to quantify working memory. The test that Brännström et al. (2012b) used to quantify working memory was an *auditory* version of the reading span task in which the examiner presented the sentences orally, which may have increased the contribution of hearing. Alternatively, the lack of a correlation between ANL and working memory can be taken to underline that ANL and speech perception in noise are different in nature. The latter account ties in with our observation that ANLs did not relate to age, hearing thresholds, and speech-in-noise perception abilities. This held in the relatively good-hearing adult sample as tested here, but was also found by Nábělek et al. (1991, 2004), Freyaldenhoven et al. (2007), Plyler et al. (2007), and Moore et al. (2011) for both normal-hearing and hearing-impaired participants. Moreover, we have not found evidence for an association between ANL and self-control abilities reported in Nichols and Gordon-Hickey (2012). However, the latter study used a self-control scale containing 36 items in contrast to the Brief Self-Control Scale with 13 items that we asked our participant to fill in.

The combined pattern of results converges on material effects being present for the ANL test with better noise tolerance and slightly better and more stable repeatability, at least numerically, for meaningful stimuli. We have also shown that activity limitations due to hearing problems and ANLs are related, especially if conversational materials are used as ANL test material. More natural speech materials can thus be used in a clinical setting as repeatability is not reduced compared to more standard materials. We aim to conduct follow-up research to investigate whether ecologically valid test materials – such as the conversational speech material used in this study – can be used to improve the predictive power of the ANL test for hearing aid success, relative to more standardized speech materials.

## Author Contributions

XK, GD, and EJ designed the experiment; XK and GD prepared the speech materials; XK conducted the experiment and analyzed the data; XK wrote the paper supported by input from GD, AG, and EJ; GD developed the computerized test procedure.

## Conflict of Interest Statement

The authors declare that the research was conducted in the absence of any commercial or financial relationships that could be construed as a potential conflict of interest.
